# Exploring Factors Associated With the Work Hours of Attending Physicians Working in Hospitals

**DOI:** 10.34172/ijhpm.2022.6242

**Published:** 2022-04-25

**Authors:** Tsung-Hsien Yu, Ying-Hui Hou, Hui-Yi Hsu, Ray-E Chang

**Affiliations:** ^1^Department of Health Care Management, National Taipei University of Nursing and Science, Taipei, Taiwan.; ^2^Department of Health Industry Management, School of Healthcare Management, Kainan University, Taoyuan, Taiwan.; ^3^Department of Operations Management, Ten-Chan General Hospital, Taoyuan, Taiwan.; ^4^Institute of Health Policy and Management, College of Public Health, National Taiwan University, Taipei, Taiwan.

**Keywords:** Work Hours, Work Hour Structure, Attending Physician, Hospital-Employed Physician, Hospital Characteristics, Specialty

## Abstract

**Background:** Long work hours for physicians not only harm the health of physicians, but also endanger patient safety. Compared with resident physicians, attending physicians—especially hospital-employed attending physicians—assume more responsibilities but has not gotten enough attention. The purpose of this study was to explore whether a hospital’s geographic location and emergency care responsibility might influence the number of hours worked.

**Methods:** The respondents of 2365 attending physicians from 152 hospitals in the 2018 survey of Taiwan physician work hours were used as the data source. The total work hour per week and its components, the regular scheduled shift and three types of on-call shifts, were used as outcome variables. Hospital geographic location and emergency care responsibility were the independent variables. The multilevel random effect model was employed to examine the study objective after adjusting for clinical specialty, hospital teaching status, and ownership.

**Results:** The average number of total working hours was 69.09 hours per week; the regular scheduled shift was account for 75% of total work hours. The results showed the total work hours were only varied by the level of hospital’s emergency care responsibility. However, the results also demonstrated the hours of duty shifts were varied by hospital’s geographic location and emergency care responsibility. The results of the multilevel random effect model revealed that the hospital’s emergency care responsibility was the factor consistently associated with attending physician’s work hour, no matter the total work hours or its composition.

**Conclusion:** In this study, we explored how a hospital’s location and its level of emergency care responsibility were associated with physicians’ work hours for each type of shift. Our findings offer an opportunity to review the rationality of physician workforce allocation, and financial incentives and administrative measures could be the next steps for balancing the work hours of attending physicians.

## Background

**Key Messages**

** Implications for policy makers**

- Physicians’ work hours and work conditions varied among various specialties, and they also varied within the same specialties.
- Hospital characteristics and geographic location should be considered when planning the physician workforce allocation and estimation.
- To reduce the work hours of attending physicians, hospital management should make sure that shifts are adequately staffed, particularly in hospitals with higher levels of emergency care responsibility, and health authorities could develop favorable policies to assist rural hospitals or hospitals with a higher level of emergency responsibility in recruiting attending physicians in high-workload specialties.

** Implications for the public**

Long work hours for physicians not only harm the health of physicians, but also endanger patient safety. Hospital-employed attending physicians assume more responsibilities, however, their work hours have not gotten enough research attention. The results revealed that the number of work hours overall of a hospital-employed attending physician were mainly associated with a hospital’s level of emergency responsibility and the type of clinical specialty. Also, the distribution of working hours in each type of shift differs between urban and rural areas. The public should be aware of the adequacy of staffed physicians and urge health authorities and hospital administrators to face the problem of long physician work hours in order to ensure healthcare quality and patient safety.

The issue of long physician work hours has been noticed since the late 1980s.^1-5^ This issue has rapidly drawn considerable attention from physician societies of different specialties. In subsequent decades, studies found that long work hours were associated with health problems in physicians ranging from chronic conditions to acute illness to sudden incidences (eg, a cardiovascular event), and even death, also known as *karoshi* (death by overwork) in Japan. Moreover, long physician work hours also endanger patient safety and life,^6,7^ and corrodes the performance and operation of healthcare organizations as well.^8-10^ Such adverse consequences have drawn concern from the public and also led the physicians, researchers, and the public to call for regulation of the work hours of physicians, mainly focused on resident physicians.^11-13^

 In addition to resident physicians, there are also attending physicians and research fellows who are employed in hospitals, and the work hours of attending physicians and research fellows have not attracted sufficient attention in previous research. Unlike resident physicians and research fellows, attending physicians usually lead medical teams: They lead resident physicians, nursing staff, pharmacists, and other medical staff in offering care to patients. They take ultimate responsibility for the actions of the medical team; therefore, in addition to working their regular duty hours, they can also be contacted by the on-duty staff seeking an attending physician’s medical orders if patients under the care of his or her medical team have sudden or urgent medical care needs during the attending physician’s non-working hours (such as vacations or during the night). In most cases, the attending physicians usually give their orders over the telephone. But when the situation becomes critical, most attending physicians will rush back to the hospital to take over the treatment because of their responsibilities. In other words, during their free time they also need to be alert to unexpected urgent situations for their patients, which could be considered an extending of their work time. Attending physicians may also assume teaching and research duties in academic affiliations and thus extend their role beyond their clinical work.

 Hospital-employed attending physicians have less autonomy in scheduling their work hours than their peers in independent practice. Moreover, in order to maintain the 24-hour, year-round continuous care to hospital inpatients, these physicians have to take turns working during different time periods in a day and on different non-work days such as weekends and holidays. Their clinical as well as teaching and research duties are rigorously defined by the hospitals that employ them. Sometimes, these attending physicians may assume organizational administrative duties to assist the hospital management. Accordingly, a complex shift system was developed for hospital-employed physicians, including a regular schedule shift, standby in hospital, a first-line on-call shift (outside hospital with return obligation), and a second-line on-call shift (outside hospital without return obligation). Since hospital attending physicians have less autonomy, their work schedules are more rigid and regulated. Hospitals are a public good and they play an important role in modern society. Putting the right resources in the right place will help improve the lives and health outcomes of residents. Therefore, the health authorities might assign certain hospitals to be responsible for certain special tasks (eg, trauma center), which might increase physician work hours.

 Therefore, the working hours of hospital attending physicians should receive more research attention, and understanding the factors associated with work hours would be beneficial for preventing burnout.^1,3-5^

 Studies on the work hours of attending physicians are few, and the existing studies show that the work hours of different specialties vary substantially.^14^ This could rationally be explained by the level of clinical responsibility and the conditions (characteristics) of the patients they work with. If a specialty does not involve direct care of patients, as is the case with radiologists or clinical pathologists, for example, their work could mainly be arranged in a fixed or hourly-based shift. Moreover, physicians in specialties that include caring for patients with higher acuity or requiring more intensive monitoring tend to spend more time in direct care and be assigned more on-call duties to take care of sudden or urgent incidences, such as obstetricians and neurosurgeons, which results in longer work hours. However, not only is the number of studies limited, but the studies^14,15^ are also mostly focused on the total work hours rather than further investigating the associations between total work hours and the number of hours spent working different types of shifts.

 In our previous study,^16^ we investigated attending physician work hours among various specialty and hospital characteristics. The results revealed that the work hours varied by specialty and hospital characteristics, and we also found that variation in the type of work hours existed.^16^ In the literature, previous studies have already found that the variations in physician work hours not only exist among specialties, but also exist within a specialty.^14,17^ Why does such a phenomenon exist? Geographic location might account for some of the variations. Physicians practicing in rural areas tend to work longer hours than their counterparts in urban areas.^16^ Organizational characteristics could be another factor. Without physician colleagues, physicians tended to work longer hours.^18^ Evidence found in research on the relationship between organizational characteristics and the work hours of other hospital professionals might warrant further research on their physician colleagues.^19,20^ As healthcare resources are a type of public good, health authorities might assign different care responsibilities to healthcare providers in order to maximize the effectiveness of healthcare resources in regional planning, for example by assigning isolation and responding hospitals for communicable diseases, drug addiction treatment and alternative treatment institutions, and children’s preventive healthcare institutions.

 Therefore, the purpose of this study was to explore whether a hospital’s level of urbanicity and level of emergency responsibility were associated with the number of work hours for each type of shift, taking the data from a national survey in Taiwan that we conducted in 2018.

## Methods

###  Study Design

 A three-level study design—clinical specialty, hospital, and contextual level—was adopted in this study to account for the nested data structure.

###  Data Source

 Data for this study were abstracted from the results of a survey of physicians’ work hours in Taiwan in 2018.^16^ This survey was conducted from June 15, 2018, to September 30, 2018, and all accredited hospitals were recruited. The study population of the 2018 physician work hours survey included the representative attending physicians and resident physicians in each medical specialty of each hospital. An invitation letter was sent to all accredited hospitals, and all participant hospitals were asked to appoint a representative physician in each clinical specialty whose work hours were representative of his/her department to fill out the questionnaire. The following information was collected, including the department and hospital, and the respondent’s demographic information, job title, seniority, and the average work hours of each of the respondent’s shifts in the past 3 months. A total of 152 hospitals agreed to participate in this study, and 2365 attending physicians returned the questionnaire to us. In this study, we only used the data of the attending physicians for achieving the purposes of our study.

###  Variables Definition 

 The number of weekly work hours, the level of urbanicity of a hospital, and the hospital’s level of emergency care responsibility were the major variables in this study, which are elaborated on below.

####  Dependent Variable: Weekly Work Hours

 The number of total weekly work hours was used as the dependent variable. The 2018 survey of Taiwan physician work hours defined the weekly work hours as including the regular scheduled shift and three types of on-call shifts: in-house on-call shifts, at-home on-call service shifts, and at-home standby shifts.^16^ The length of these four types of work shifts and total work hours per week were used as the dependent variables in this study.

####  Independent Variables

 A hospital’s level of urbanicity and the hospital’s level of emergency care responsibility in the healthcare system were used as the independent variables in this study.

 The geographic location of each hospital was used to classify the hospital’s urbanization level, according to the definition of urbanization published by Taiwan’s National Health Research Institutes. All 365 townships in Taiwan were classified into seven clusters based on the following indicators: population density (people/km^2^), proportion of people with a college degree or above, proportion of people over 65 years old, proportion of people who are agriculture workers, and the number of physicians per 100 000 people. Residential areas located in cluster 1 were categorized as urban; others were categorized as rural.

 In Taiwan, hospitals with emergency care departments are assigned one of three levels of emergency care responsibility: basic, intermediate, and advanced, and are required to earn the accreditation of hospital emergency care ability from the Ministry of Health and Welfare (MoHW). Referring to the list of 2017 hospital emergency care accreditation, we categorized our participant hospitals into four levels: advanced, intermediate, basic, and none (those without any emergency care responsibility).

####  Covariates

 A hospital’s teaching status, ownership, and medical specialties were used as covariates in this study. Participant hospitals were sorted into three types of hospital ownership: government-owned, not-for-profit, and private hospitals, the latter including proprietary and cooperative hospitals. As for medical specialty, there are 23 medical specialties officially certified by Taiwan’s MoHW, which were used for specialty classification in this study. In our previous study,^16^ we did a hierarchical cluster analysis for categorizing, based on work hours spent in different shifts as well as total work hours. The number of clusters was decided by the researchers based on the Cubic Clustering Criterion and rationality of the cluster numbers. Finally, the 23 medical specialties were classified into four groups, with the workload of cluster 1 described as mild and physicians in cluster 4 described as extremely exhausted, as compared to their peers.

###  Statistical Analysis

 All statistical analysis was performed by SAS 9.4. Frequency and percentage were used to describe the distributions of our responses. The means and standard deviations of physician work hours in different shifts and in totality were calculated. Because our data are not distributed normally, we used the Mann-Whitney U and Kruskal-Wallis tests to test the differences in the work hours of each work shift with respect to various hospital characteristics. A three-level random intercept model was used for achieving the study purpose. We used variance inflation factor and conditional index to avoid collinearity issues. Further, a stratified analysis was also conducted for comparing work hour differences between urban and rural hospitals under the same hospital emergency care level and clinical specialty. A subgroup analysis for clinical clusters was also conducted.

## Results

Table 1 shows the distribution of our sample. A total of 2365 physicians from 152 hospitals responded this survey. 642 (27.15%) physicians’ hospitals were located in urban areas. Around 80% of respondent physicians served in 78 hospitals with advanced or intermediate emergency care responsibilities. Most of our respondent physicians are employed in medium and large-sized hospitals, or teaching hospitals, or public and not-for-profit hospitals. As for clinical specialty, the physicians who belonged to specialty cluster 3 returned the largest number of questionnaires (42%), and physicians in clusters 2 and 4 each returned close to 25% of all surveys, respectively. From the point of view of participant hospital characteristics, 21.05% of them were located in urban areas, about half of them were hospitals with an advanced or intermediate level of emergency care responsibility, more than 50% of them were teaching hospitals, and the majority of participant hospitals were nonprofit.

**Table 1:** Sample Distribution

	**Hospitals** **(n = 152)**	**Physicians** **(n = 2365)**
Urbanicity		
Urban	32 (21.05)	642 (27.15)
Rural	120 (79.95)	1723 (72.85)
Emergency care responsibility		
Advanced	19 (12.50)	725 (30.66)
Intermediate	59 (38.82)	1146 (48.46)
Basic	34 (22.37)	267 (11.29)
None	40 (26.32)	227 (9.60)
Teaching		
Yes	81 (53.29)	1866 (78.90)
No	71 (46.71)	499 (21.10)
Ownership		
Public	48 (31.58)	842 (35.60)
Nonprofit	71 (46.71)	1399 (56.62)
Private	33 (21.71)	184 (7.78)
Physician specialty		
Specialty cluster 1	-	189 (7.99)
Specialty cluster 2	-	600 (25.37)
Specialty cluster 3	-	1001 (42.33)
Specialty cluster 4	-	575 (24.31)

Data are expressed as mean (standard deviation).

Table 2 shows the number and percentage of work hours for each shift broken down according to hospital characteristics. The average number of total working hours was 69.09 hours per week; the average number of hours worked on a regular scheduled shift was 51.23, which accounted for almost 75 of total work hours; and the average number of work hours of the three types of on-call shifts (in-house on-call shift, at-home on-call shift, and at-home standby shift) were 8.13, 7.29, and 2.44, which accounted for 11.77%, 10.55%, and 3.53%, respectively. The results also revealed that the hospital’s geographic location was not associated with the number of total work hours, but that the composition of work hours did vary between urban and rural locations: Rural physicians spent less time on regular scheduled shifts, and instead had longer work hours on on-call shifts. Furthermore, the results also showed that physicians who were employed by hospitals with a higher level of emergency care responsibility, teaching hospitals, and public or nonprofit hospitals had longer work hours. However, this did not mean that the work hours of each type of shift were longer. For example, although physicians who were employed by hospitals with advanced emergency responsibility had the longest total work hours and the longest work hours for regular scheduled work shifts, the work hours spent on on-call shifts were sometimes shorter than for other groups. Regarding the proportion of work hours for each shift, the results revealed that for physicians who served in urban areas, hospitals with no emergency care responsibilities, and private hospitals, the number of work hours spent on regular scheduled shifts occupied a higher proportion of their total number of work hours. We also found that physicians who served in rural areas, in hospitals with higher emergency care responsibilities, and in public hospitals spent more work hours dealing with emergency cases (on at-home on-call service shifts and at-home standby shifts).

**Table 2:** The Work Hours of Each Shift Among Hospital Characteristics

	**Total Work ** **Hours**	**Scheduled Work ** **Shift**	**In-House On-Call ** **Shift**	**At-Home On-Call Service Shift**	**At-Home Standby Shift**
All	69.09 (21.42)/100	51.23 (13.48)/74.14	8.13 (10.59)/11.77	7.29 (11.45)/10.55	2.44 (5.69)/3.53
Urbanicity	.5487^a^	.0001^a^	.0310^a^	.0113^a^	.0004^a^
Urban	68.67 (19.61)/100	52.91 (13.41)/80.10	7.04 (9.92)/9.12	5.90 (9.98)/7.22	2.82 (6.08)/3.56
Rural	69.24 (22.06)/100	50.60 (13.46)/77.14	8.54 (10.80)/10.80	7.80 (11.91)/9.28	2.30 (5.54)/2.77
Emergency care responsibility	<.0001^b^	<.0001^b^	<.0001^b^	<.0001^b^	<.0001^b^
Advanced	73.10 (17.94)/100	56.43 (12.03)/79.74	6.44 (9.98)/7.81	6.85 (10.12)/8.26	3.38 (6.23)/4.19
Intermediate	71.63 (21.03)/100	51.14 (11.75)/75.47	9.50 (10.83)/11.84	8.53 (12.68)/9.83	2.45 (6.11)/2.86
Basic	60.11 (22.83)/100	45.36 (14.39)/80.20	7.20 (9.89)/10.04	6.49 (10.97)/8.32	1.06 (3.23)/1.45
None	53.99 (22.54)/100	41.95 (16.73)/82.08	7.70 (11.16)/11.27	3.34 (7.83)/5.06	1.00 (2.54)/1.59
Teaching status	<.0001^a^	<.0001^a^	.1546^a^	.0001^a^	<.0001^a^
Yes	70.98 (20.53)/100	52.82 (12.61)/77.86	7.86 (10.24)/12.27	7.58 (11.55)/7.93	2.72 (6.06)/1.94
No	62.01 (23.17)/100	45.28 (14.90)/77.97	9.14 (11.74)/9.83	6.19 (11.01)/8.93	1.41 (3.86)/3.27
Ownership	<.0001^b^	<.0001^b^	.0005^b^	<.0001^b^	.0005^b^
Public	72.17 (20.25)/100	51.49 (12.75)/74.79	9.05 (10.72)/11.45	9.00 (12.91)/10.59	2.62 (5.91)/3.17
Nonprofit	69.07 (21.20)/100	52.19 (13.29)/79.26	7.71 (10.59)/9.64	6.82 (10.82)/8.19	2.34 (5.48)/2.91
Private	55.07 (22.75)/100	43.01 (15.28)/82.83	6.97 (9.65)/10.43	2.79 (5.95)/4.03	2.30 (6.21)/2.70

Data are expressed as mean (standard deviation)/percentage of total work hours. ^a^*P* value, Mann-Whitney U test; ^b^*P* value, Kruskal-Wallis test.

Table 3 shows the results of our multilevel analysis. We found that the level of emergency care responsibility, a physician’s clinical cluster, and hospital ownership were all associated with total work hours. As for individual types of shifts, emergency care responsibility, clinical cluster, and hospital ownership were associated with work hours. Looked at from another perspective, work hour differences among clinical clusters existed for all work shifts; the work hour differences among physicians at hospitals with different emergency care responsibility levels were on regular scheduled shifts or at-home on-call service shifts or at-home standby shifts.

**Table 3:** The Results of Multilevel Regression

	**Total Work Hours**		**Scheduled Working**		**In-House On-Call**		**At-Home On-Call Service**		**At-Home Standby**	
	**β (SE)**	***P*** ** Value**	**β (SE)**	***P*** ** Value**	**β (SE)**	***P*** ** Value**	**β (SE)**	***P*** ** Value**	**β (SE)**	***P*** ** Value**
Physician specialty (ref = cluster1)										
Cluster 2	5.93 (1.42)	<.0001	3.91 (0.97)	<.0001	-0.01 (0.78)	.9935	1.93 (0.90)	.0310	0.25 (0.47)	.5860
Cluster 3	17.22 (1.35)	<.0001	3.41 (0.93)	.0002	7.64 (0.75)	<.0001	5.30 (0.85)	<.0001	0.85 (0.44)	.0551
Cluster 4	25.51 (1.42)	<.0001	5.60 (0.97)	<.0001	7.76 (0.78)	<.0001	10.17 (0.90)	<.0001	1.98 (0.47)	<.0001
Urbanicity (ref = rural)										
Urban	-1.50 (2.69)	.5776	0.16 (1.93)	.9347	-0.34 (1.11)	.7587	-2.01 (0.94)	.0322	0.16 (0.43)	.7113
Emergency care responsibility (ref=none)										
Advanced	15.65 (4.35)	.0003	12.49 (2.99)	<.0001	-1.09 (1.88)	.5627	2.00 (1.68)	.2348	2.99 (0.79)	.0001
Intermediate	15.82 (3.78)	<.0001	8.32 (2.59)	.0013	2.23 (1.63)	.1699	3.62 (1.47)	.0141	2.13 (0.69)	.0022
Basic	2.44 (3.41)	.4744	2.83 (2.33)	.2232	-2.02 (1.49)	.1755	1.93 (1.39)	.1627	0.38 (0.66)	.5633
Teaching (ref = none)										
Yes	-1.17 (3.03)	.7001	0.16 (2.09)	.9406	-2.22 (1.28)	.0826	0.58 (1.13)	.6065	0.02 (0.52)	.9639
Ownership (ref = private)										
Public	8.07 (3.49)	.0207	1.57 (2.37)	.5067	2.72 (1.52)	.0741	4.29 (1.40)	.0022	-1.04 (0.67)	.1190
Nonprofit	1.95 (3.40)	.5666	0.18 (2.32)	.9367	0.84 (1.48)	.5732	2.34 (1.36)	.0869	-1.45 (0.65)	.0255

Abbreviation: SE, standard error.

 Finally, Table 4 shows the results of stratified analysis under the same clinical cluster and the same emergency care responsibility level, the difference in work hours between urban physicians and rural physicians. We found that differences existed in total work hours between urban and rural physicians who were employed by hospitals with basic emergency care responsibility in clinical specialty clusters 1 (borderline significant), 2, and 4. We also found that the urban-rural difference in work hours in hospitals with a basic level of emergency care responsibility were caused by the difference between the scheduled working shift and in-house on-call shift. Further, the results also showed that urban-rural work hour differences existed in a certain shift, but such differences did not have a significant impact on the total work hours: for example, in the case of the regular scheduled working shift in cluster 1 and the at-home on-call service shift in cluster 3 in hospitals with advanced emergency care responsibility, and in the case of the at-home standby shift in cluster 4 in hospitals with no emergency care responsibility.

**Table 4:** Stratified Analysis Results: A Comparison of Urban-Rural Physician Work Hours in Hospitals With the Same Level of Emergency Care Responsibility and Among Physicians With the Same Clinical Specialty

	**Cluster 1**			**Cluster 2**			**Cluster 3**			**Cluster 4**		
	**β**	**SE**	***P*** ** Value**	**β**	**SE**	***P*** ** Value**	**β**	**SE**	***P*** ** Value**	**β**	**SE**	***P*** ** Value**
Total work hours												
Advanced	2.95	5.51	.5958	0.29	2.73	.9150	-1.49	2.91	.6085	-2.25	5.57	.6865
Intermediate	-8.18	6.97	.2478	-5.32	4.31	.2188	-3.01	4.47	.5022	3.00	4.56	.5120
Basic	18.89	8.18	.0820	-15.82	6.83	.0265	-11.13	8.41	.1898	-20.83	9.43	.0339
None	19.20	9.79	.3002	7.45	13.24	.5792	4.50	8.62	.6033	7.08	8.12	.3928
Scheduled working								
Advanced	6.11	3.08	.0550	0.81	2.30	.7266	0.03	2.32	.9912	5.67	3.90	.1476
Intermediate	-0.97	5.01	.8481	-2.64	2.22	.2366	1.14	2.44	.6406	3.89	3.09	.2094
Basic	17.98	7.58	.0767	-9.50	5.32	.0826	-4.66	6.31	.4617	-12.71	6.74	.0675
None	7.85	7.58	.4888	1.99	7.67	.7974	-1.82	7.13	.7996	0.52	5.99	.9321
In-house on-call												
Advanced	-1.51	1.75	.3932	-1.04	0.68	.1318	0.27	1.59	.8649	-3.13	2.32	.1780
Intermediate	-1.85	1.92	.3430	-0.55	2.00	.7827	-4.37	2.13	.0408	0.67	3.02	.8245
Basic	-0.43	2.94	.8901	-4.44	2.52	.0876	-3.51	3.30	.2898	-3.96	5.51	.4772
None	5.70	3.35	.3382	9.48	10.23	.3635	5.55	3.97	.1667	4.28	5.01	.4021
At-home on-call service												
Advanced	-0.49	2.19	.8254	0.79	1.66	.6359	-3.29	1.68	.0513	-3.17	3.06	.3022
Intermediate	-4.53	3.66	.2232	-1.79	1.76	.3077	-0.11	2.09	.9569	-3.89	3.38	.2518
Basic	0.52	0.47	.3306	-2.82	3.23	.3879	-4.12	3.83	.2863	-4.05	4.82	.4071
None	4.57	2.39	.3071	-4.85	6.17	.4394	-0.41	2.46	.8691	0.77	2.29	.7397
At-home standby												
Advanced	-2.12	2.04	.3047	-0.58	1.76	.7417	1.54	1.04	.1377	-2.18	1.84	.2383
Intermediate	-1.11	1.76	.5331	0.15	1.02	.8859	-0.36	0.87	.6776	2.38	1.53	.1213
Basic	0.67	0.93	.5098	-0.20	0.64	.7516	1.94	1.18	.1048	-0.14	0.84	.8662
None	0.93	1.17	.5714	-0.23	0.66	.7340	0.58	0.59	.3261	2.98	1.32	.0346

Abbreviation: SE, standard error.

## Discussion

 Attending physicians in Taiwan are usually employed in hospitals or clinics (the latter includes physicians who are self-employed). Physicians who are employed in hospitals not only offer clinical services, but also teach and conduct research; once they become managers, they have to take on administrative responsibilities as well. In contrast to attending physicians working in hospitals, offering clinical services is the main work of physicians who work in clinics. To the best of our knowledge, only a few studies have looked at hospital-employed attending physicians in order to determine which factors are associated with their total number of work hours and their number of work hours on each type of shift. Our results showed the hospital’s level of emergency service responsibility, physicians’ clinical specialty, and type of hospital ownership played important roles in attending physicians’ work hours, and although the difference in work hours between physicians working in urban and rural areas was not significant, in the stratification analysis, we still found there was an association, especially in the distribution of hours worked on each type of shift. In other words, even though the total work hours are not different between urban and rural areas, a variation in workload as broken down by shift type might still exist.

 Regarding our findings, there are some issues worth discussing. First, why did physicians who were employed by hospitals with a higher level of emergency care responsibility have longer work hours? This might be attributable to the fact that hospitals with a higher level of emergency care responsibility were responsible for caring for patients with more serious and emergent conditions, which resulted in heavier workloads for their physicians. Similarly, public and nonprofit hospitals in Taiwan are expected to be more social welfare–oriented and may therefore assume more social responsibility. Thus, these hospitals may schedule more at-home on-call shifts for their attending physicians in order to cover unexpected emergent cases arising during non-regular working times such as nighttime, weekends, or holidays. Therefore, it was also expected that physicians who were employed by public and nonprofit hospitals had longer work hours on at-home on-call service shifts.

 Second, was the unequal distribution of physicians associated with the difference in the distribution of work hours for each kind of shift between urban and rural areas? As in many countries, healthcare resources in Taiwan, including the workforce, are not distributed equally between urban and rural areas. Most healthcare resources are concentrated in urban areas. The most updated MoHW statistics reveal that the median of physicians working in urban areas was 22.28 per 10 000 people; however, the median in rural areas was 14.75,^21^ showing that the workforce in less urbanized areas was relatively insufficient. When rural-dwelling patients have urgent medical needs, they will still be sent to the nearest hospital for immediate treatment, regardless of the hospital’s staffing level. Therefore, physicians who worked at rural hospitals spent more work hours on in-house on-call shifts than the physicians who worked at hospitals in areas that were more urban.

 Third, why were the attending physicians’ work hours longer in Taiwan than in some other countries? Previous studies usually focused on total work hours, not hours worked on different types of shifts, and therefore it’s difficult for us to compare all of our findings with them. The total weekly work hours per physician was the only one we could use for comparison. Our findings showed the average total weekly work hours per physician was 69.1, which was similar to previous studies, which showed that the hospital-employed attending physicians in Eastern countries (eg, Japan^22-24^ and Taiwan^25^) had longer work hours than their Western counterparts (eg, the United States^14,26,27^ and the United Kingdom^28^). The variance might be due to the differences in healthcare systems and culture. Our findings also revealed that surgery-related specialties had longer weekly work hours than others, which was also confirmed by other existing studies.^14,29^ As we mentioned above, physicians in specialties involving caring for patients with higher acuity or requiring more intensive monitoring tend to spend more time in direct care and to be assigned more on-call duties to take care of sudden or urgent incidences, and therefore it is not unexpected that their work hours were longer. But our findings can differentiate between the hours of regular working shift and on-call shifts, which was a shortcoming of the previous studies. Regarding the effects of level of urbanicity on the work hours, our findings showed that the higher the degree of urbanization, the higher the proportion of hours spent on a regular working shift, which was similar to McGrail et al,^30^ and we also found that the higher the degree of urbanization, the longer the total work hours, which was not consistent with Steinhaeuser et al (Germany)^31^ and Weeks and Wallace (US),^32^ but was similar to Leu et al (Taiwan).^33^

 A fourth area of concern has to do with the survey method employed. In the existing literature, two approaches can be found for investigating physicians’ work hours: a direct approach and an indirect approach. Surveying individual physicians directly is commonly used.^1,3,17,18^ While this approach can access the sampled individuals equally, the major problem is the low response rate. Moreover, the uneven distribution of the responding sample, eg, among specialties or among settings, is another issue. Both issues could seriously endanger the representativeness of the sample. On the other hand, an indirect approach can be adopted to survey the representative physicians through the hospital administrative system, which is the approach we chose in this study. The advantage of this approach is that once the consent of participation is obtained from a hospital, the responses of all medical specialties in the hospital can be almost guaranteed and obtained. This could alleviate the low response rate and uneven distribution among specialties.^25,34^ Still, two potential problems might arise. One is the representativeness of the participating hospitals; the other is the representativeness of physicians chosen for each specialty in a hospital. Either approach has its strengths and weaknesses. Since the difference in work hours among specialties was important information and needs to be adjusted in this study, we chose the indirect approach to collect more comprehensive representativeness in terms of physician specialty.

 In this study we undertook two measures to lessen the adverse impacts resulting from the weaknesses of the indirect approach. Since this national project was initiated and supported by the MoHW, hospital consent could be expected through the official assistance from the MoHW, which could thus reduce the risk of not having a representative sample of hospitals. Moreover, in order to minimize the risk of a lack of representativeness of physicians chosen for each specialty in an individual hospital, we held ten orientations throughout Taiwan, from north to south and from west to east, and invited each accredited hospital to assign several physician heads and a human resource director to attend one of these orientations. In addition to the detailed explanation on the definitions of the four work shifts, we also explained during the orientations how to choose a representative physician and verify the questionnaire was filled out by the representative physicians. The representative physician was to be selected in a formal meeting of physicians in his/her specialty, and the work hour questionnaire filled out by the representative physician was to be reviewed in another formal meeting of physicians in his/her specialty. We believe these measures can resolve the possible adverse impacts of the indirect approach to a great extent and consider the indirect approach to be the optimal and most efficient one for us.

 Finally, how can our findings be applied to balancing the difference in work hours? Ideally, under the same conditions, a physician’s work hours should be very similar, no matter the number of work hours or the percentage of hours spent on each kind of shift. The findings of this study offer evidence of how physicians spent their time on different duty shifts in different conditions. Hospital managers could reduce unnecessary physician shift assignments or increase the physician workforce on certain shifts in some specialties in order to improve the efficiency and effectiveness of care. For health authorities and policy-makers, regulation and financial incentives (eg, subsidies for physician salary) could be used to balance the work hour difference. However, both hospital administrators and policy-makers must note that a similar total number of work hours does not necessarily represent the same workload, as the workload distribution can vary between regular scheduled shifts and on-call shifts.

###  Advantages and Limitations

 In this study we have adopted several measures to increase the representativeness and the accuracy of the information on work hours, but there are limitations in the study that need to be addressed. Firstly, recall bias would be unavoidable for a questionnaire survey. Firstly, recall bias is unavoidable in a questionnaire survey. The work hours of each shift for each attending physician exist in the personnel database in every hospital, so theoretically, participating hospitals could provide this data through their information technology (IT) system, and it would also be possible to offer more detailed information (eg, the case number of each shift) to measure the workload of each attending physician. However, we found that requesting such data diminished the willingness of hospitals to participate in this study, because providing such data would add to the already heavy workload of their IT departments. Therefore, after considering the pros and cons of both approaches, we decided to adopt the questionnaire survey to maximize the number of participating hospitals. Secondly, the representativeness of respondents. Although we asked hospitals or department chiefs to appoint a representative physician to complete our survey, potential bias exists if they did not appoint such a physician. Lastly, there are also several factors that might affect the number of work hours, such as the average seniority of a physician and the age of the department, and the department size, among others. However, this information was not collected because of the survey method we used. Lack of these variables might diminish the validity of our results.

## Conclusion

 In this study we focused on how hospital’s characteristics were associated with attending physicians’ work hours and work conditions. Through our findings, we not only discovered the association between hospital characteristics and the work hours of attending physicians, but also understood how the work hours varied among each type of shift. The findings of this study offer empirical evidence for future physician workforce planning, and additional actions such as further regulation of physician work hours and financial incentives (eg, subsidies for physician salaries or reimbursement policies) could be used for balancing out the differences in work hours for attending physicians.

## Ethical issues

 The Institutional Review Board of the National Taiwan University Hospital approved the protocol that was used in the present study (protocol #201911106W). Informed consent was acquired from all respondents, and all experiments were performed in accordance with relevant guidelines and regulations.

## Competing interests

 Authors declare that they have no competing interests.

## Authors’ contributions

 Conception and design: THY and REC. Acquisition of data: REC. Analysis and interpretation of data: THY, YHH, HYH, and REC. Drafting of the manuscript: THY and YHH. Critical revision of the manuscript for important intellectual content: REC. Statistical analysis: THY and YHH. Administrative, technical, or material support: HYH. Supervision: REC. All authors contributed to read and approve the final version.

## Funding

 This study was supported by the Taiwan Ministry of Health and Welfare (Grant No: M06A7439).
